# Associations Between Abortion Services and Acceptance of Postabortion Contraception in Six Indian States

**DOI:** 10.1111/j.1728-4465.2015.00039.x

**Published:** 2015-12-08

**Authors:** Sushanta K. Banerjee, Sumit Gulati, Kathryn L. Andersen, Valerie Acre, Janardan Warvadekar, Deepa Navin

**Affiliations:** ^1^ Senior Director, Ipas Development FoundationP.O. Box 8862, Vasant ViharNew Delhi 110 057India; ^2^ Assistant Manager, Ipas Development FoundationP.O. Box 8862, Vasant ViharNew Delhi 110 057India; ^3^ Manager, Ipas Development FoundationP.O. Box 8862, Vasant ViharNew Delhi 110 057India; ^4^ Director, Research and Evaluation, Ipas Development FoundationP.O. Box 8862, Vasant ViharNew Delhi 110 057India; ^5^ Senior Advisor, Research and Evaluation, IpasChapel HillNC 27516; ^6^ Advisor, Research and Evaluation, IpasChapel HillNC 27516

## Abstract

Women receiving induced abortions or postabortion care are at high risk of subsequent unintended pregnancy, and intervals of less than six months between abortion and subsequent pregnancy may be associated with adverse outcomes. This study highlights the prevalence and attributes of postabortion contraceptive acceptance from 2,456 health facilities in six major Indian states, among 292,508 women who received abortion care services from July 2011 through June 2014. Eighty‐one percent of the women accepted postabortion contraceptive methods: 53 percent short‐term, 11 percent intrauterine devices, and 16 percent sterilization. Postabortion contraceptive acceptance was highest among women who were aged 25 years and older, received first‐trimester services, received induced abortion, attended primary‐level health facilities, and had medical abortions. Doctors receiving post‐training support were more likely to offer contraceptives, but no association was observed between such support and acceptance of IUDs or sterilization. Comprehensive service‐delivery interventions, including ensuring availability of skilled providers and contraceptive commodities, offering clinical mentoring for providers, identifying and addressing provider bias, and improving provider counseling skills, can increase postabortion contraceptive acceptance and reduce unintended pregnancy.

Postabortion contraception—the provision of contraceptive counseling and services at the time of, or immediately following, induced abortion or postabortion care—is an essential element of abortion care (Postabortion Care Consortium Community Task Force 2002; WHO 2012). Worldwide, approximately 70 million of the 205 million pregnancies yearly end in abortion. Of these, 42 million are induced abortions (WHO 2011). India alone accounts for 6.4 million induced abortions and approximately 4 million spontaneous abortions each year, most performed in unsafe conditions (Duggal and Ramachandran 2004; Banerjee and Andersen 2012; Banerjee, Andersen, and Warvadekar 2012) and often without any contraceptive counseling or services (Banerjee and Manning 2010). Although women request abortions for various reasons, including contraceptive failure, limiting or delaying pregnancy, family opposition to pregnancy, and poverty (Ravindran and Balasubramanian 2004), unintended pregnancy is the primary reason cited (Banerjee and Andersen 2012). Termination of pregnancy itself provides evidence of unintended pregnancy and unmet need for contraception (Cleland, Harbison, and Shah 2014).

Integrating contraceptive services into other health services provides a unique opportunity to reduce unmet need for contraception (Solo et al. 1999; Malarcher and Polis 2014); this is particularly true for women seeking abortion care. Ovulation has been documented as occurring as early as six days after abortion (Lahteenmaki and Luukkainen 1978; Schreiber, Ratliffe, and Creinin 2011) in comparison with one month after childbirth, making the timely provision of contraception imperative. Also, women who have had an abortion are at potential risk of having a subsequent abortion (Makhlouf et al. 2014), and an interval of less than six months between an induced or incomplete abortion and a subsequent pregnancy is significantly associated with adverse pregnancy outcomes (Conde-Agudelo et al. 2005; Conde-Agudelo, Rosas-Bermudez, and Ana 2007; Bigelow and Bryant 2015). Research from various settings indicates that provision of postabortion contraception after uterine evacuation can increase women's contraceptive acceptance and intention to use a modern method after discharge from a health facility, and thus can also reduce subsequent unplanned pregnancies and abortions (Solo et al. 1999; Lema and Mpanga 2000; Medina et al. 2001; Johnson et al. 2002; Goodman et al. 2008; McDougall et al. 2009; Zhu et al. 2009). On the basis of this evidence, several international agencies endorsed the 25 September 2009 consensus statement to ensure postabortion contraceptive counseling and services through integration of family planning and postabortion contraception (FIGO et al. 2009). Postabortion contraception is an essential part of comprehensive abortion care (CAC), which takes into account women's health needs, personal circumstances, and ability to obtain services (Hyman and Kumar 2004). In India, the new national training and service-delivery guidelines for CAC (MOHFW 2010) and strategic guidelines for reproductive, maternal, newborn child, and adolescent health (MOHFW 2013) stipulate provision of contraceptive counseling, services, and postabortion follow-up visits to reinforce contraceptive use.

Limited data are available in India regarding contraceptive counseling and services and the quality of postabortion contraceptive care; however, existing data suggest that while women want to adopt a postabortion contraceptive method (Ganatra and Hirve 2002; Agarwal, Chauhan, and Modi 2007), many receive inadequate or no postabortion services (Jejeebhoy, Zavier, and Kalyanwala 2010). Facility-based surveys conducted in three states recorded a prevalence of postabortion contraceptive acceptance ranging from 39 percent in Meghalaya (Navin et al. 2011b) and 54 percent in Chhattisgarh (Navin et al. 2011a) to 79 percent in Uttarakhand (Banerjee et al. 2009). A study in Maharashtra revealed significant variations in counseling and contraceptive acceptance by age. Women aged 20 years and younger are less likely to receive postabortion contraceptive counseling and methods than women over age 20 (Ganatra and Hirve 2002). Evidence for India of postabortion contraception after incomplete abortion is even more limited. A prospective study among women receiving care for postabortion complications in ten districts and medical college hospitals in Madhya Pradesh found that almost three-fourths of women who presented with abortion-related complications received no postabortion contraception (Banerjee and Andersen 2012). A qualitative study in Uttar Pradesh found no uniformity in postabortion contraceptive counseling, service provision, or documentation among public, private, and informal-sector providers (Banerjee and Manning 2010). The study identified many factors that may inhibit the acceptance of postabortion contraception, including misconceptions and individual biases among providers, the tendency of providers to promote sterilization and intrauterine devices (IUDs) to women who have undergone a free abortion, lack of provider accountability for counseling women before and after an abortion, and the private-sector perception that modern contraceptive methods do not provide much revenue. Providers are often unaware of the need to offer postabortion contraception or have misconceptions and biases that prompt them to provide only short-term methods after induced or incomplete abortions. Unlike general contraceptive prevalence, which is often influenced by the individual characteristics of couples, postabortion contraception appears to be strongly influenced by the abortion provider.

There is little research on the service-delivery correlates of prevalence and methods of postabortion contraception in India. Such information could help identify appropriate provider, site, and policy responses to increase the accessibility and quality of postabortion contraceptive services. In this article, we describe postabortion contraceptive acceptance and identify which characteristics of abortion service delivery are associated with overall acceptance and type of method among a large cohort of women receiving abortion-care services in six Indian states. We compare these associations before and after implementation of a post-training provider-support intervention to see whether such training had a significant effect on postabortion contraceptive acceptance and method mix.

### SETTING

#### Comprehensive Abortion Care Model

In India, Ipas Development Foundation (IDF) collaborates with state health systems to reduce maternal mortality and morbidity resulting from unsafe abortion by implementing a comprehensive abortion care model to improve access to safe abortion services at all levels of health facilities (primary, secondary, and tertiary) in rural and urban areas. The CAC model is integrated into the health system, and interventions are characterized by training, facility preparation, post-training provider follow-up and mentoring, monitoring of performance data, community-level education, and advocacy. In line with the government's training and service-delivery guidelines (MOHFW 2010), medical doctors receive training on abortion care and management and treatment of postabortion care, including contraceptive counseling and service provision. Nursing staff (who are legally not allowed to provide induced abortion services) are trained in postabortion care, infection management, contraceptive counseling and service delivery, and documentation. At the facility level, efforts are made to ensure that an adequate supply of contraceptive methods is maintained, and providers are encouraged to keep a sufficient stock of contraceptives in the room where abortion care is provided. At the provider level, post-training follow-up was given to all CAC providers trained after June 2011, to ensure that they could offer need-based individual support to resolve clinical or administrative issues identified during facility visits and other contacts. Providers trained before July 2011 did not receive need-based individual follow-up support between July 2011 and June 2014. At the community level, IDF staff oriented outreach workers in abortion and postabortion care, including postabortion contraceptive methods and safe sources of supply.

#### Intervention Sites

This study was conducted in six Indian states where IDF collaborated with state governments between July 2011 and June 2014: Maharashtra from the western region, Madhya Pradesh and Uttarakhand from the central region, Rajasthan from the northern region, and Bihar and Jharkhand from the eastern region. These states contain 33 percent of the country's population and account for an estimated 27 percent of the total number of annual induced abortions.

#### Ongoing Monitoring

To track the quality of abortion services and contraception provision after abortion, data were collected from each intervention facility at least every six months. On the basis of these data, IDF staff worked with government officials to ensure the availability of contraceptive supplies at district and state levels, and collaborated with individual providers to improve postabortion contraceptive provision (including comprehensive counseling, skilled providers, and a range of commodities) to ensure that women can obtain their preferred method.

### METHODS

#### Tools and Data Management

As a routine protocol of the comprehensive abortion care intervention, IDF collected individual case records of women seeking abortion or postabortion-care services, using a structured abortion logbook developed in accordance with the Indian Medical Termination of Pregnancy (MTP) Act of 1971 (Government of India 2004). These logbooks, maintained by CAC-trained medical doctors or nursing staff, contain details of services provided. The following logbook variables were used in this analysis: woman's age (individual-level characteristics); gestational age of pregnancy, diagnosis (induced or incomplete abortion), abortion method, provider identification, procedure date, and postabortion contraceptive acceptance and method mix (service-delivery characteristics). In addition, post-training provider support, recorded by IDF staff at the time of each contact, was used to indicate exposure to the post-training support intervention. All data were entered into the IDF global monitoring and evaluation database and imported into Stata 12 for data validation and analysis.

#### Data Analysis

Analysis was performed on the individual case records of 292,508 women who received abortion services in the three-year period from July 2011 to June 2014 at 2,456 intervention facilities in six Indian states. The primary outcome variables of interest for this study were prevalence of postabortion contraception and contraceptive method mix. Prevalence was determined by whether women accepted a contraceptive method at the time of the abortion or immediately after an abortion procedure or treatment for a postabortion complication, as recorded in the logbook. For the purpose of this analysis, missing data for contraceptive acceptance (11 percent) were treated as "given no contraception." The method mix was further categorized as short-term (pills, condoms, and injectables), long-acting reversible (IUD), and permanent (female sterilization).

Descriptive statistics are reported for both categorical and continuous variables. Categorical variables were analyzed using frequencies and percentages. For continuous variables, means are reported. To assess factors influencing the prevalence of postabortion contraception, we explored unadjusted and adjusted associations between contraceptive acceptance and age, clinical diagnosis, gestational age of pregnancy, abortion method, facility level and sector, post-training mentoring support received by provider who performed the abortion, year of abortion procedure, and state-level contraceptive prevalence rate (CPR). We classified the abortion procedure into three time categories: Year 1 (July 2011 to June 2012), Year 2 (July 2012 to June 2013), and Year 3 (July 2013 to June 2014). To assess possible associations between CPR and postabortion contraceptive acceptance, we classified the six intervention states into high CPR (greater than 60 percent), medium CPR (40–60 percent), and low CPR (less than 40 percent) regions, based on the state-specific rate. A similar analysis was conducted to explore attributes influencing the acceptance of short-term and long-acting contraceptive methods after abortion through unadjusted and adjusted associations with service-delivery characteristics of interest.

All unadjusted associations were analyzed using frequencies and percentages, while adjusted analyses were conducted using logistic regressions presented with adjusted odds and p-values. Two separate logistic regression models were used for the outcome variables "women received any postabortion contraception" (Yes=1, No=0) and "method mix of postabortion contraceptives" (permanent method=2, IUD=1, short-term=0), after controlling for age, method of abortion, facility level and type, provider receiving post-training support, and year of procedure.

### RESULTS

During the three-year study period, 292,508 women received abortion services from 2,456 IDF-supported health facilities in six states. As shown in Table 1, 37 percent of women were aged 24 years or younger. Almost two-thirds of the women (65 percent) requested induced abortion services and the remaining 35 percent received treatment for incomplete abortion or postabortion complications. A large majority received abortion services in the first trimester (91 percent) and only 2 percent in the second trimester. Gestational age was missing for 7 percent of cases, primarily among women who presented with incomplete abortion. Women predominantly underwent surgical abortion (83 percent), including manual/electric vacuum aspiration (MVA/EVA) (76 percent) or dilation and curettage (7 percent), while 15 percent received medical abortion (MA) using mifepristone and misoprostol. Because the IDF intervention was primarily focused on the public health system, the majority of abortion cases were recorded at public facilities (82 percent),

**TABLE 1 Characteristics of 292,508 women who received abortion services at 2,456 intervention sites, six states in India, July 2011 to June 2014**

| Characteristic | n | % |
|---|---|---|
| Age | | |
| ≤ 24 | 106,942 | 36.6 |
| > 24 | 180,900 | 61.8 |
| Missing | 4,666 | 1.6 |
| Mean age, among nonmissing | 26.2 | |
| Gestation | | |
| First trimester | 266,017 | 90.9 |
| Second trimester | 6,391 | 2.2 |
| Missing | 20,100 | 6.9 |
| Gestation, among nonmissing (mean) | 7.9 | |
| Diagnosis | | |
| Induced | 188,518 | 64.5 |
| Incomplete/Other | 101,899 | 34.8 |
| Missing | 2,091 | 0.7 |
| Facility sector | | |
| Public | 239,516 | 81.9 |
| Private | 43,333 | 14.8 |
| NGO/Trust | 9,659 | 3.3 |
| Facility level | | |
| Secondary and tertiary | 167,953 | 57.4 |
| Community health center | 47,837 | 16.4 |
| Primary health center/Private clinic | 76,718 | 26.2 |
| Abortion method | | |
| MVA | 196,775 | 67.3 |
| EVA | 25,840 | 8.8 |
| MA | 42,361 | 14.5 |
| D&C | 21,561 | 7.4 |
| Missing/Other | 5,971 | 2.0 |
| Year of abortion procedure | | |
| Year 1 (July 2011–June 2012) | 37,731 | 12.9 |
| Year 2 (July 2012–June 2013) | 106,051 | 36.3 |
| Year 3 (July 2013–June 2014) | 148,726 | 50.9 |
| State | | |
| Madhya Pradesh | 98,381 | 33.6 |
| Maharashtra | 77,564 | 26.5 |
| Rajasthan | 46,867 | 16.0 |
| Bihar | 38,879 | 13.3 |
| Jharkhand | 20,805 | 7.1 |
| Uttarakhand | 10,012 | 3.4 |

MVA = Manual vacuum aspiration. EVA = Electric vacuum aspiration.
MA = Medical abortion. D&C = Dilation and curettage.
NOTE: All variables are significant ($p < 0.001$).
SOURCE: MTP register (logbooks).

with only 18 percent at private sector and NGO facilities. More than half of the abortion cases were recorded at secondary- and tertiary-level hospitals (57 percent), including medical colleges and district and subdistrict hospitals, while the remaining 43 percent were recorded at primary-level facilities, including primary health centers (PHCs) (11 percent), community health centers located in rural areas (16 percent), and private clinics (15 percent).

As shown in Table 2, 81 percent of women received contraception immediately after abortion care, while 19 percent left without a method. Contraceptive pills were the most common form of contraception (33 percent). Just over half of the women received short-term methods (53 percent), compared with 28 percent who accepted IUDs (11 percent) and female steriliza-

**TABLE 2 Percentage of women receiving postabortion contraception, by method, among 292,508 abortion clients, six states in India, July 2011 to June 2014**

| | n | % |
|---|---|---|
| Received postabortion contraception | | |
| Yes | 236,955 | 81.0 |
| No | 55,553 | 19.0 |
| Method | | |
| Pills | 96,826 | 33.1 |
| Condoms | 58,853 | 20.1 |
| Female sterilization | 47,876 | 16.4 |
| IUDs | 33,212 | 11.4 |
| Injectables | 188 | 0.06 |
| None | 55,553 | 19.0 |
| Short-term method[a] | 155,867 | 53.3 |
| IUD and female sterilization | 81,088 | 27.7 |

[a]Condoms, pills, and injectables.
NOTE: All variables are significant ($p < 0.001$).
SOURCE: MTP register (logbooks).

tion (16 percent). The prevalence of postabortion contraceptive acceptance was not uniform across the study period, as shown in Figure 1. Among women who received abortion services before the addition of post-training provider support (baseline), 36 percent received a contraceptive method at primary- and at secondary- or tertiary-level facilities. At the beginning of the post-training support (in July 2011), contraceptive acceptance increased to 68 percent, whereas overall prevalence increased to 88 percent after almost three years of post-training support (June 2014). Postabortion contraceptive acceptance at primary health facilities was significantly higher than at secondary- and tertiary-level hospitals throughout the intervention period. Overall, contraceptive prevalence was positively correlated with time ($R^2 = 0.64$; $\beta = 0.79$), demonstrating significant improvement as post-training support became more routine.

**FIGURE 1 Percentage of women receiving abortion care who accepted contraception, total and by facility level, six states in India, July 2011 to June 2014**

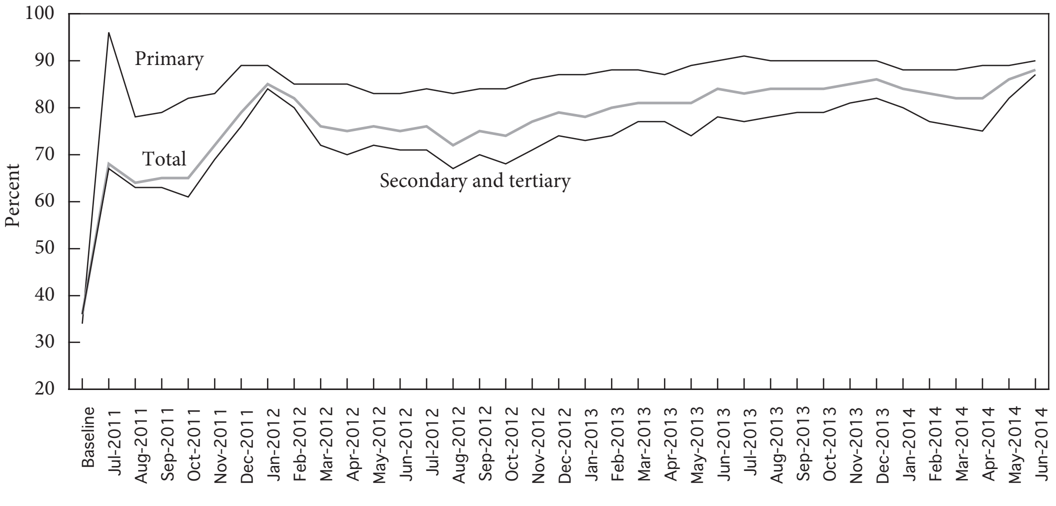

We examined the likelihood of acceptance of postabortion contraception by individual and health-system indicators through unadjusted and adjusted associations (Tables 3 and 4). Women aged 25 years or older have slightly increased odds of receiving a postabortion contraceptive method, compared with younger women (Table 3, 84 percent; Table 4, AOR = 1.3). Diagnosis and gestational age of pregnancy have even stronger associations with contraceptive acceptance in both unadjusted and adjusted models. Women presenting for induced abortion had nearly four times higher odds of receiving postabortion contraception than women presenting for incomplete abortion (AOR=3.9). Similarly, women who received first-trimester abortions were more likely to receive any contraception (84 percent; AOR= 1.7) than women who received second-trimester abortions (62 percent). Women who received medical abortion had a higher likelihood (AOR=1.5) of receiving any contraception. Health-facility sector had the strongest association with postabortion contraceptive acceptance. Women who received abortion services at an NGO-run facility had much higher odds of receiving any contraception (AOR=4.7) than women who had an abortion at a public-sector facility. Further, women who sought care from

**TABLE 3 Prevalence of postabortion contraception by characteristics of women and service provision among 292,508 abortion clients, six states in India, July 2011 to June 2014**

| Characteristics of women and service provision | n | % |
|---|---|---|
| Received postabortion contraception | 236,955 | 81.0 |
| Age | | |
| ≤ 24 | 84,175 | 78.7 |
| > 24 | 151,161 | 83.6 |
| Gestation | | |
| Second trimester | 3,976 | 62.2 |
| First trimester | 224,055 | 84.2 |
| Diagnosis | | |
| Incomplete/Other | 66,693 | 65.5 |
| Induced | 169,279 | 89.8 |
| Abortion method | | |
| Medical | 37,299 | 88.1 |
| Surgical | 196,520 | 80.5 |
| Facility sector | | |
| Public | 188,498 | 78.7 |
| Private | 43,333 | 90.6 |
| NGO/Trust | 9,659 | 95.2 |
| Facility level | | |
| Secondary and tertiary | 127,119 | 75.7 |
| Community health center | 39,780 | 83.2 |
| Primary health center/Private clinic | 70,056 | 91.3 |
| Provider received post-training support | | |
| No | 106,869 | 75.2 |
| Yes | 130,086 | 86.5 |
| Year of abortion procedure | | |
| Year 1 (July 2011–June 2012) | 28,413 | 75.3 |
| Year 2 (July 2012–June 2013) | 83,754 | 79.0 |
| Year 3 (July 2013–June 2014) | 124,788 | 83.9 |
| State-level CPR | | |
| Low | 56,404 | 94.5 |
| Moderate | 116,222 | 74.8 |
| High | 64,329 | 82.9 |

CPR= Contraceptive prevalence rate.
NOTE: All variables are significant ($p < 0.001$).

**TABLE 4 Adjusted logistic regression analysis of characteristics of women and service provision influencing acceptance of postabortion contraception among 263,989 abortion clients, six states in India, July 2011 to June 2014**

| Characteristics of women and service provision | Received any postabortion contraception (AOR[a]) |
|---|---|
| Age | |
| ≤ 24 (r) | |
| > 24 | 1.3*** |
| Gestation | |
| Second trimester (r) | |
| First trimester | 1.7*** |
| Diagnosis | |
| Incomplete/Other (r) | |
| Induced | 3.9*** |
| Abortion method | |
| Medical (r) | |
| Surgical | 1.5*** |
| Facility sector | |
| Public (r) | |
| Private | 0.8*** |
| NGO/Trust | 4.7*** |
| Facility level | |
| Secondary and tertiary (r) | |
| Community health center | 1.3*** |
| Primary health center/Private clinic | 1.9*** |
| Provider received post-training support | |
| No (r) | |
| Yes | 1.2*** |
| Year of abortion procedure | |
| Year 1 (July 2011–June 2012) (r) | |
| Year 2 (July 2012–June 2013) | 1.2*** |
| Year 3 (July 2013–June 2014) | 1.7*** |
| State-level CPR | |
| Low (r) | |
| Moderate | 0.3*** |
| High | 0.3*** |

***Significant at $p < 0.001$. AOR= Adjusted odds ratio. (r) = Reference category.
CPR= Contraceptive prevalence rate.
[a]AOR adjusted for age, gestation, diagnosis, abortion method, facility level and type, CPR, year of procedure, and mentoring support.
NOTE: Above model does not include missing cases; number of cases used for analysis is 263,989.
SOURCE: MTP register (logbooks).

private-sector facilities had decreased odds of receiving postabortion contraception, compared with women at public-sector facilities (AOR=0.8). The type of health facility where abortions were performed was also associated with contraceptive acceptance. Women who were served at lower-level facilities had higher odds of receiving postabortion contraception than women at higher-level facilities. For example, 91 percent of women at primary-level facilities received contraception, compared with 76 percent of women at secondary- or tertiary-level hospitals. Women receiving abortion care from providers who underwent post-training mentoring had slightly higher odds of receiving postabortion contraception than women receiving abortion care from providers with training only (AOR=1.2). Women who received abortion services any time during the third year of the intervention had 1.7 times higher odds of receiving a postabor-

**TABLE 5 Acceptance of short-term methods, IUDs, or female sterilization among 236,955 postabortion contraception acceptors, by characteristics of women and service provision, six states in India, July 2011 to June 2014**

| Characteristics of women and service provision | Short-term method[a] | | IUD | | Female sterilization | |
|---|---|---|---|---|---|---|
| | n | % | n | % | N | % |
| Women received contraception by method | 155,867 | 53.3 | 33,212 | 11.4 | 47,876 | 16.4 |
| Age | | | | | | |
| ≤ 24 | 64,044 | 60.0 | 11,421 | 10.7 | 8,710 | 8.1 |
| > 24 | 90,709 | 50.1 | 21,610 | 12.0 | 38,842 | 21.5 |
| Gestation | | | | | | |
| Second trimester | 2,885 | 45.1 | 312 | 4.9 | 779 | 12.2 |
| First trimester | 146,187 | 55.0 | 32,001 | 12.0 | 45,867 | 17.2 |
| Diagnosis | | | | | | |
| Incomplete/Other | 55,419 | 54.4 | 6,329 | 6.2 | 4,945 | 4.9 |
| Induced | 998,870 | 53.0 | 26,700 | 14.2 | 42,692 | 22.7 |
| Abortion method | | | | | | |
| Medical | 33,269 | 78.5 | 2,160 | 5.1 | 1,870 | 4.4 |
| Surgical | 120,736 | 49.5 | 30,655 | 12.6 | 45,129 | 18.5 |
| Facility sector | | | | | | |
| Public | 119,113 | 49.7 | 28,129 | 11.7 | 41,256 | 17.2 |
| Private | 31,245 | 72.1 | 3,590 | 8.3 | 4,424 | 10.2 |
| NGO/Trust | 5,509 | 57.0 | 1,493 | 15.5 | 2,196 | 22.7 |
| Facility level | | | | | | |
| Secondary and tertiary | 72,862 | 43.4 | 22,475 | 13.4 | 31,782 | 18.9 |
| Community health center | 31,191 | 65.2 | 3,436 | 7.2 | 5,153 | 10.8 |
| Primary health center/Private clinic | 51,814 | 67.5 | 7,301 | 9.5 | 10,941 | 14.3 |
| Provider received post-training support | | | | | | |
| No | 66,222 | 46.6 | 16,671 | 11.7 | 23,976 | 16.9 |
| Yes | 89,645 | 59.6 | 16,541 | 11.0 | 23,900 | 15.9 |
| Year of abortion procedure | | | | | | |
| Year 1 (July 2011–June 2012) | 16,688 | 44.2 | 4,054 | 10.7 | 7,671 | 20.3 |
| Year 2 (July 2012–June 2013) | 53,563 | 50.5 | 11,888 | 11.2 | 18,303 | 17.3 |
| Year 3 (July 2013–June 2014) | 85,616 | 57.6 | 17,270 | 11.6 | 21,902 | 14.7 |
| State-level CPR | | | | | | |
| Low | 39,272 | 65.8 | 7,241 | 12.1 | 9,891 | 16.6 |
| Moderate | 88,090 | 56.7 | 11,934 | 7.7 | 16,198 | 10.4 |
| High | 28,505 | 36.8 | 14,037 | 18.1 | 21,787 | 28.1 |

CPR= Contraceptive prevalence rate.
[a]Condoms, pills, and injectables.
NOTE: All variables are significant ($p < 0.000$).
SOURCE: MTP register (logbooks).

tion contraceptive than women who received abortion services in the first year. The associations between state-level CPR and postabortion contraceptive acceptance were surprising: women in states with moderate and high CPRs had one-third the odds of postabortion contraceptive acceptance compared with women in states with low CPRs.

Characteristics of women and service provision influencing postabortion contraceptive method mix among the women who accepted any contraceptive method are presented in Tables 5 and 6. Approximately one-fourth of acceptors received either IUDs (11 percent) or sterilization (16 percent). As was seen with overall method acceptance, younger women and women presenting for second-trimester abortions had lower odds of receiving IUDs and sterilization than women aged 25 years or older and women presenting in the first trimester. Furthermore, women presenting for induced abortions had greater odds of receiving IUDs (AOR=2.8) and sterilization (AOR=5.1) than women who received treatment for incomplete or postabortion

**TABLE 6 Adjusted logistic regression analysis of factors influencing acceptance of IUDs and female sterilization among 222,965 postabortion contraception acceptors, six states in India, July 2011 to June 2014**

| Characteristics of women and service provision | AOR[a] IUD | AOR[a] Female sterilization |
|---|---|---|
| Age | | |
| ≤ 24 (r) | | |
| > 24 | 1.5*** | 3.5*** |
| Gestation | | |
| Second trimester (r) | | |
| First trimester | 2.0*** | 1.7*** |
| Diagnosis | | |
| Incomplete/Other (r) | | |
| Induced | 2.8*** | 5.1*** |
| Abortion method | | |
| Medical (r) | | |
| Surgical | 4.4*** | 8.4*** |
| Facility sector | | |
| Public | | |
| Private | 0.9*** | 0.7*** |
| NGO/trust | 0.7*** | 0.7*** |
| Facility level | | |
| Secondary and tertiary (r) | | |
| Community health center | 0.5*** | 0.6*** |
| Primary health center/Private clinic | 0.5*** | 0.6*** |
| Provider received post-training support | | |
| No (r) | | |
| Yes | 1.0 | 1.1*** |
| Year of abortion procedure | | |
| Year 1 (July 2011–June 2012) (r) | | |
| Year 2 (July 2012–June 2013) | 1.0 | 0.8*** |
| Year 3 (July 2013–June 2014) | 1.2*** | 0.8*** |
| State-level CPR | | |
| Low (r) | | |
| Moderate | 0.8*** | 0.8*** |
| High | 2.8*** | 3.3*** |

***Significant at $p < 0.001$. (r) = Reference category. CPR = Contraceptive prevalence rate.
[a]AOR = Odds ratio adjusted for age, gestation, diagnosis, abortion method, facility level and type, CPR, year of procedure, and mentoring support.
NOTES: This model does not include missing cases; number of cases used for analysis is 222,965.
SOURCE: MTP register (logbooks).

complications. Contrary to the findings of the previous model of any contraceptive acceptance, surgical abortion was strongly associated with acceptance of long-acting or permanent methods: women who received abortion services with any surgical method were 4.4 times more likely to receive IUDs and 8.4 times more likely to receive sterilization than women who had a medical abortion. Sector and type of facility were also associated with method mix of postabortion contraception. Lower IUD acceptance was reported for women receiving abortion services at community health centers (7 percent; AOR=0.5) and primary health centers/private clinics (10 percent; AOR=0.5); similarly, sterilization was less often reported for women who received abortion services at community health centers (11 percent; AOR=0.6) and primary health centers/private clinics (14 percent; AOR=0.6). While there was no association between post-training mentoring of providers and acceptance of IUDs, there was some association with sterilization (AOR=1.1). State-level CPR was strongly associated with method mix. Women from states with high CPRs were more likely to receive IUDs (18 percent; AOR=2.8) and sterilization (28 percent; AOR=3.3) than women from states with low CPRs.

## DISCUSSION

### Prevalence and Method Mix

We found a high level of postabortion contraception (81 percent), with 53 percent of women accepting short-term spacing methods, namely, contraceptive pills, condoms, and injectables, and 28 percent accepting long-acting or permanent methods, namely IUDs and female sterilization. This mix of methods highlights substantial improvements in method choice. Furthermore, postabortion contraceptive acceptance increased from 68 percent in the first month of the intervention to 88 percent at the end, suggesting that program improvements and post-training provider support visits to health facilities likely helped integrate postabortion contraception into comprehensive abortion care. Integration of these reproductive health services had long been missing (Urban Health Initiative 2010), despite being integral to successful provision of postabortion contraception.

### Factors Influencing Contraceptive Acceptance and Choice of Contraceptive Method

Results of bivariate and multivariate analyses found associations between postabortion contraceptive acceptance and women's age, facility level and type, gestational age of pregnancy, diagnosis, abortion method, and post-training mentoring support.

#### *Age*

More than one-third of women who received abortion services were below age 25 and had lower contraceptive acceptance, especially for long-acting methods like IUDs, than women aged 25 or older. It is likely that the established positive association between parity and contraceptive acceptance continues to influence decisions of both providers and clients.

#### *Diagnosis: Induced Abortion and Postabortion Care*

Stronger associations were noted by diagnosis. Women who had an induced abortion in the facility had a much higher acceptance of any method, especially of long-acting methods or sterilization, than women seeking care for postabortion complications. These findings are in line with earlier studies in India (Banerjee et al. 2014) that show providers' disinclination to offer contraception, and more specifically long-acting methods, immediately after postabortion care, primarily because of fear of infection (Banerjee and Manning 2010).

#### *Medical and Surgical Abortion*

Associations were found between abortion method and contraceptive acceptance. Whereas a larger proportion of medical abortion clients received postabortion contraception, significantly fewer medical abortion clients received long-acting methods like IUDs and sterilization, compared with surgical abortion clients. In contrast with other studies (Banerjee et al. 2011; Kalyanwala, Acharya, and Zavier 2012), overall contraceptive acceptance after medical abortion was much higher in our study as a result of short-term method acceptance in conjunction with dispensing the first dose of medical abortion drugs. Thus, providing condoms and hormonal methods such as pills, injectables, and implants, which can be offered on the first day of medical abortion in accordance with WHO guidelines (WHO 2009), appears to increase contraceptive acceptance following medical abortion. However, women who had surgical abortion were more likely to accept long-acting methods than those who had medical abortion. This variation may be explained by the fact that concurrent sterilization and IUD insertion are easily feasible after a surgical procedure (Kalyanwala, Acharya, and Zavier 2012), whereas providers often ask women receiving medical abortion to wait until their menstrual cycle resumes (Banerjee et al. 2011; Kalyanwala, Acharya, and Zavier 2012). Implants are still not available in India, and injectables are available only in a limited number of private-sector clinics. This results in missed opportunities for women to receive effective methods, because the likelihood of a return visit is low (Banerjee et al. 2011).

#### *Sector and Type of Health Facility*

Higher contraceptive acceptance was observed in NGO and private-sector health facilities than in public health facilities. Although earlier studies had observed a similar trend for NGO-run health facilities (Banerjee and Manning 2010), a high prevalence of postabortion contraception in the private sector is a new phenomenon (Aich et al. 2011). Interestingly, a contrary trend was observed for long-acting or permanent methods: among women who received any method, private-sector clients had lower acceptance of long-acting or permanent methods than did public-sector and NGO clients. As demonstrated in earlier studies (Billings et al. 1999; Banerjee and Manning 2010), we observed a higher prevalence of contraceptive acceptance at primary-level health facilities than at secondary- and tertiary-level hospitals. However, once again this association was reversed with regard to long-acting or permanent methods, acceptance of which was significantly higher at secondary- and tertiary-level health facilities. Overall prevalence was low at higher-level facilities, primarily because of lack of access to short-term spacing methods (such as condoms and pills) in the procedure room where abortion services are provided. Offering contraceptive counseling and methods in the same location where abortion services are offered increases the chances of women accepting a postabortion contraceptive method (Solo et al. 1999; Billings and Benson 2005; McDougall et al. 2009). Provision of long-acting or permanent methods at the primary level and at private facilities is significantly lower, probably because of a lack of skilled providers trained in IUD insertion and female sterilization, and inadequate infrastructure to support these procedures.

#### *Post-training Mentoring of Providers*

We found that providers who trained between July 2011 and June 2014 and received long-term post-training support provided better contraceptive services than their counterparts who trained before July 2011 and received no post-training support. A similar variation can be observed by time of intervention, which shows that contraceptive acceptance improved over time. This intervention was designed so that newly trained providers received support to improve individual performance and to ensure quality provision of CAC services, including postabortion contraceptives. However, post-training support was weakly associated with acceptance of long-acting contraceptive methods, again most likely because providers had no training in IUD insertion and female sterilization. Future interventions should consider offering more effective methods of contraception by training providers in IUD insertion and sterilization.

#### *Strengths and Limitations*

The strength of this study lies in the large sample of 292,508 women who received abortion services at public, private, and NGO facilities in six states in India—the first of its kind in the country to investigate postabortion contraceptive acceptance. However, our findings should be viewed within the context of the study's limitations. Data are taken from facility-based service statistics (MTP logbooks) that collected very limited information in accordance with the guidance of the Indian MTP Act of 1971. Inclusion of other individual, household, or state-level characteristics could strengthen our analysis of correlates of postabortion contraception, quality and content of counseling, and available methods. In addition, only one method was recorded for each woman; information on provision of condoms along with other methods for preventing sexually transmitted infections (STIs) is not available. We reported contraceptive acceptance at the time of abortion care, but we do not have data on contraceptive continuation over time. Recent multicountry analyses suggest that contraceptive discontinuation accounts for up to 50 percent of current unmet need (Jain et al. 2013). In a study in India, Kalyanwala, Acharya, and Zavier (2012) found a 5 percent and 8 percent discontinuation rate by 24 weeks among women who opted for reversible contraceptive methods after surgical and medical abortion, respectively. Discontinuation rates were highest (36 percent and 38 percent, respectively) among women who opted for hormonal methods. Additionally, our findings are based on six Indian states where IDF is working to improve access to safe abortion services and cannot be generalized to the whole of India. Although abortion continues to be available from untrained and illegal providers, this study captures only the formal sector where abortion is provided by a CAC-trained doctor. In addition, our data are drawn from facilities where IDF is supporting CAC services, and no comparison group of public facilities without IDF intervention is available. Finally, we are unable to compare the contraceptive method women wanted with the method they actually received. Despite these limitations, most of the study's findings related to postabortion contraception are in line with other research in India.

### CONCLUSION

Postabortion contraception, a critical component of comprehensive abortion care, includes contraceptive counseling and provision of methods. Integration of services increases postabortion contraceptive acceptance at all levels of health facilities. This analysis has shown service-delivery associations with postabortion contraceptive acceptance, including post-training support to abortion providers. However, crucial impediments to integration have also been identified, namely, treatment of incomplete abortion, lack of skilled or trained providers for providing long-acting contraceptive methods in certain facilities, lack of availability of newer contraceptive methods such as injectables and implants, and lack of technical clarity on contraception and method mix after medical abortion.

Postabortion contraception is still being treated as part of family planning services rather than as a component of maternal health. This perspective ignores the potential risk of maternal mortality and morbidity associated with short pregnancy intervals and results in a missed opportunity for intervention. It is essential to strengthen the family planning component of comprehensive abortion care services in order to prevent unintended pregnancy. Approximately 10 million Indian women who receive abortion-related services every year can be provided with effective contraceptive methods by improving the available method mix, providing effective counseling, and ensuring contraceptive choice. The findings of this study can be used to scale up this initiative across all states of India.

Efforts to decentralize CAC services to primary health facilities are promising. Primary-level facilities now provide abortion care and other reproductive health services to the majority of rural women in India, and as such have the potential to increase the prevalence of postabortion contraceptive acceptance. Although this study demonstrates a high prevalence of post-abortion contraception at primary-level facilities, further gains may be made by introducing more effective methods like IUDs, injectables, and sterilization, currently restricted because of a lack of skilled providers, poor infrastructure, and insufficient commodities. Policies are needed to expand women's choice of modern methods by introducing implants and improving the availability of injectables in the public sector. India's national government and state governments should ensure sufficient resources for commodities and supplies of all modern methods. Future research should incorporate women's perspectives on postabortion contraception to ensure greater access and informed choice.

### ACKNOWLEDGMENTS

We thank Iqbal H. Shah, John Cleland, K.G. Santhya, and Vinoj Manning for their critical reviews and insights. Data presented, statements made, and views expressed are solely the responsibility of the authors.
